# Evaluation of a guidelines implementation intervention to reduce work disability and sick leaves related to chronic musculoskeletal pain: a theory-informed qualitative study in occupational health care

**DOI:** 10.1186/s12891-022-05234-8

**Published:** 2022-03-22

**Authors:** Ritva Horppu, Ari Väänänen, Johanna Kausto

**Affiliations:** Finnish Institute of Occupational Health, P.O Box 40, 00032 Helsinki, Finland

**Keywords:** Musculoskeletal pain, Sick leave, Guideline implementation, Intervention evaluation, Occupational health care

## Abstract

**Background:**

Guidelines for pain management and sick leave prescription were formulated and implemented in an occupational health services (OHS) in Finland to reduce work disability and sick leaves related to musculoskeletal pain. We investigated how the guidelines implementation intervention may have produced its effects, how the number of prescribed sick leave days varied before and after the launch of the guidelines, and which factors beyond physician behaviour were seen to influence sick leaves.

**Methods:**

Seventeen physicians, two occupational physiotherapists and one occupational health care nurse were interviewed. Qualitative content analysis using both inductive and deductive approaches was performed, informed by Behaviour Change Wheel and Theoretical Domains Framework. Employees’ sick leave days related to musculoskeletal disorders in 2015–2019 were drawn from the employer’s register.

**Results:**

Physicians’ guidelines adherence was facilitated by psychological capability (e.g., having relevant knowledge, remembering to engage in recommended behaviours), reflective motivation (e.g., guidelines-related behaviours regarded as central part of one’s professional role; beliefs in the positive consequences of recommended behaviours to employees and employers), and physical and social opportunities (e.g., adequate physical resources, culture of social support). Some physicians also described barriers to recommended behaviours (e.g., lack of knowledge or non-pharmacological pain treatment tools). The guidelines had served as sources of new knowledge, reminders of recommended practices and means of self-assessment. Considerable declining trend of prescribed sick leave days was detected, especially during the first years after the intervention, levelling off somewhat thereafter. OHS policies and structures were seen to enable professionals’ focusing on preventing pain-related disability and prolonged sick leaves. The decline of sickness absences was also attributed to the municipal client organization’s commitment and the employees’ positive attitudes towards the alternatives to full-time sick leave.

**Conclusions:**

The guidelines implementation intervention was found successful. The study showed the importance of social and organizational environment supporting physicians’ engagement in recommended practices.

**Supplementary Information:**

The online version contains supplementary material available at 10.1186/s12891-022-05234-8.

## Introduction

Musculoskeletal disorders (MSDs) are highly prevalent among working age population, and they result in extensive disability costs both for the individuals and the society. In Finland in 2020, 26% of the compensated days of sick leave were due to MSDs [1]. Thus, sick leave is typically granted for musculoskeletal pain symptoms, such as back, shoulder, or elbow pain. Guidelines for evaluation of the need and length of sick leaves have been called for previously in Finland [2].

Physicians in primary care, including occupational health, have a central role in managing MSD-related work disability and sick leave days [3–5]. In this study, we explored a guidelines implementation intervention aiming to prevent and reduce work disability and sick leaves related to chronic musculoskeletal pain. The intervention was carried out in a Finnish occupational health services (OHS). The OHS formulated and introduced in 2016 guidelines for pain management and sick leave prescribing concerning most common MSD diagnoses (low back pain, elbow pain and shoulder pain). Additional guidelines concerning other diagnostic categories have been introduced later. The specific aims of the guidelines were to decrease between-physician variation in sick leave prescribing, increase the use of alternatives to full-time sick leave early on, enhance pain management, and influence patients’ pain-related beliefs and attitudes.

Previous research [6–11] has identified physicians’ key pain management behaviours (e.g., diagnosing and treating pain, assessing work disability and the need for sick leave, advising patients with pain) and factors that influence these behaviours (physicians’ knowledge and attitudes, external factors). Interventions aiming to promote physicians’ engagement in behaviours recommended by pain management guidelines may affect physicians’ knowledge and attitudes, but changing actual behaviour is more difficult, especially if organizational, workplace and health care system level factors, such as resources, cultures and policies are not targeted [12–18]. In the OHS intervention, the implementation of the guidelines was supported by three short educational sessions and some coaching sessions for physicians, led by pain specialists. Knowledge of pain, prevention and treatment of pain, and need for imaging in pain disorders was delivered. An e-learning course provided knowledge of the assessment of work disability and need for sick leave, and the alternatives for full-time sick leave. In addition, the numbers of sick leave days on pain-related diagnoses were presented in monthly staff meetings. The guidelines was part of a multi-component OHS development project, aiming to prevent and reduce MSD-related work disability and sick leaves in a large client organization. Besides the guidelines, the project included education for occupational physiotherapists and occupational health nurses; direct access to physiotherapists; pain management groups for patients, led by nurses; and a media campaign about the project and its aims, targeted to the management, supervisors and employees of the client organization.

Social and health psychological knowledge is increasingly used for conducting evaluations of existing interventions [19–25]. This study was guided by the Behaviour Change Wheel (BCW) [26, 27] to characterize the OHS guidelines implementation intervention and to explore how it may have produced its effects. The BCW has proved to provide a fruitful theoretical basis to develop and evaluate interventions in health care settings [21–25]. The BCW directs intervention developers and evaluators to studying key behaviours in context and exploring comprehensively various influences of current and recommended behaviours [26]. The BCW is a synthesis of various theoretical frameworks of behaviour change and is based on a model of human behaviour, the COM-B model, which presents behaviour (B) as a result of interaction between psychological and physical capability (C), opportunity (O) provided by the physical and social environment, and reflective and automatic motivation (M). The BCW includes component-specific intervention functions (e.g., education, persuasion, environmental restructuring) and supporting policy categories (e.g., legislation, communication and marketing), through which desired behaviour can be influenced. Additionally, we applied the Theoretical Domains Framework (TDF) for a detailed analysis of the factors falling under the three COM-B components [28, 29]. Finally, we investigated which external and even unexpected factors, beyond the possible changes in physicians’ behaviours, may have influenced the numbers of MSD-related sick leaves during the intervention and the follow-up period.

We studied:

1. What was the rationale and the means of the OHS guidelines implementation intervention? Specifically, we explored the intervention designer’s and implementers’ judgments of key guidelines-related physician behaviours, identified barriers to these behaviours, and applied means to overcome the barriers.
2. How has the intervention influenced target physicians’ behaviours with pain patients? Specifically, we explored physicians’ perceptions of guidelines adherence and the intervention received, and current influences of recommended behaviours.
3. How has the number of MSD-related sick leave days prescribed in the OHS varied before and after the launch of the guidelines?
4. Which factors, in addition to physician behaviours, are likely to influence the number of pain-related sickness absences, as perceived by the OHS professionals?

## Methods

### Study context

We conducted a theory-informed qualitative evaluation [26, 27] of a partly ongoing guidelines implementation intervention, carried out in an occupational health services (OHS). The OHS employs around 150 professionals (e.g., physicians, nurses, psychologists, physiotherapists, rehabilitation counsellors). In Finland [30], licensed physicians working full-time in occupational health care must be specialists in occupational health care. Physicians working part-time in occupational health care must have taken a minimum of fifteen credits in occupational health care studies (one credit = 27 study hours). Licensed public health nurses, physiotherapists and psychologists must have taken a minimum of fifteen credits in occupational health care studies in order to be employed as occupational health care experts. Occupational health services providers may also employ other experts, such as qualified social workers, who have taken a minimum of two credits in occupational health care studies.

The OHS provides occupational health care to a large municipal organization with approximately 40,000 employees working mainly in social services and health care, education, administration, transport, and construction and maintenance. In 2020, women comprised 75% of the employees. The employees aged from 50 to 59 years were the largest age group.

### Participants

In 2019–2020, we conducted interviews with 17 physicians: the intervention head designer and three members of the OHS management (hereafter called collectively as implementers), 9 occupational physicians and 4 general practitioners. The implementers were approached by telephone and email, and they all accepted the invitation to participate. An invitation letter containing information about the study was prepared and distributed by the chief physician to all OHS physicians (approximately 40 during the time of the study), and thirteen of them agreed to participate in the interviews. On our request, the chief physician recruited later two occupational physiotherapists and one occupational health nurse to be interviewed and provide us a broader understanding of pain management in the OHS.

Participants’ (16 female, 4 male) overall working experience ranged from two to 38 years. Ten participants had worked in this OHS less than 5 years, six participants had 5–10 years of experience, three participants had 11–20 years of experience, and one participant had worked more than 20 years in this OHS.

### Data collection and analysis

For the purposes of the qualitative study, thirteen interviews altogether, five in small groups (2 to 3 participants) and eight individual interviews were conducted in 2019–2020 either at the OHS or via Teams-meetings (after the onset of the COVID-pandemic). No other persons were present in the interviews. Semi-structured interview protocols (see Additional file 1), based on the COM-B model and prior information of the OHS guidelines implementation intervention, were developed for different parties and modified iteratively over the course of the study as new relevant themes emerged. Subsequent questions were posed to follow up participants’ accounts of experiences and perceptions. Participants were also encouraged to raise issues that they considered important beyond the predefined topics. Interviews with the implementers lasted approximately 90 min, and 45–60 min with other participants, and were audio recorded and transcribed verbatim. Transcripts were not returned to participants for comment and/or correction. No repeat interviews were carried out. Field notes were made after the interviews. The first author (female, PhD, specialized researcher) and the second author (male, PhD, research professor) conducted the interviews. They are social psychologists with long tenure as researchers of work disability and occupational health, comprehensive experience in conducting interviews and good knowledge on occupational health care. No relationships were established between the interviewers and the participants prior to the study. The interviewers introduced themselves briefly during the interviews (name, position at the Finnish Institute of Occupational Health). The researchers do not identify biases or prior assumptions that would have influenced the interviews. Being familiar to this field, but not being physicians themselves, the researchers were less likely to influence the way the participants approached the questions in the interviews.

The OHS provided us with register data on MSD-related sickness absence days prescribed in the OHS before and after the launch of the guidelines (covering the period from January 1, 2015 to December 31, 2019).

The interview data were analyzed by qualitative content analysis, using both inductive and deductive approaches [31, 32]. Inductive analysis was conducted to identify which physician behaviours the implementers considered essential for achieving the aims of the guidelines. Transcripts were read repeatedly to achieve an overall understanding of the data. During the detailed reading, implementers’ accounts of desired behaviours were extracted and categorized into key guidelines-related behaviours.

The COM-B components and included 14 TDF domains were used as a coding framework [27] to identify implementers’ retrospective perceptions of factors influencing key physician behaviours during the initiation of the intervention. Similar analysis was conducted to identify target physicians’ perceptions of current influences of the recommended behaviours. The analysis proceeded from detailed reading of the data to the extraction of themes of influencing factors. Identified themes were mapped into relevant TDF domains and further into appropriate COM-B components. A theme was assessed significant and included in the results if it was mentioned at least in a third of target physicians’ interviews, or if conflicting views were expressed concerning the theme, i.e., it was described as a barrier by some physicians and as a facilitator by others [33].

Furthermore, the BCW was used as a coding framework to identify the component-specific means through which the intervention was intended to change physician behaviours [27]. The means described by the implementers were extracted from the transcripts and linked to appropriate BCW intervention functions.

Inductive content analysis was conducted to identify which factors, in addition to physician behaviours, were perceived by all interviewees as influencing the number of MSD-related sick leave days. Accounts of influences were extracted from the transcripts and categorized into different types of factors.

All authors read the interview transcripts and discussed the general paths of coding, but the data was coded by one author (RH). No software was used to manage the data. To ensure the accuracy of coding, published guides and relevant studies were used as reference material [22,23,27,33]. All data could be classified according to the existing codes and no new categories of barriers and facilitators or intervention functions were developed. Findings were discussed with other authors, and final results were agreed by all authors. Participants were not asked to provide feedback on the findings.

Finally, for descriptive purposes, MSD-related sick leave days of the employees in 2015–2019 were drawn from the registers of the employer and presented as the total number of sick leave days per month and also per diagnostic category: low back pain (ICD-10 M54), shoulder pain (ICD-10 M75), elbow pain (ICD-10 M77.1).

## Results

Implementers’ perceptions of key guidelines-related physician behaviours, barriers to the behaviours, and applied means to overcome the barriers.

Key guidelines-related behaviours, identified from the implementers’ interviews, were diagnosing and treating pain, assessing work disability and need for sick leave, using alternatives to full-time sick leave, and advising patients with pain (see Table 1).

**Table 1:** Key guidelines-related physician behaviours

Behaviour	Sub-behaviours
**Diagnosing and treating pain**	Conducting anamnesis and clinical examination and providing a diagnosis of a specific pathology or non-specific low back pain, elbow pain or shoulder pain Using comprehensive tools for pain treatment, especially non-pharmacological tools Referring to evidence-based adjunct treatments (such as physiotherapy) or specialists Detecting high-risk disability cases (occupational physicians)
**Assessing work disability and need for sick leave**	Using evidence-based methods in work disability assessment Taking into account the complexity of pain and work disability, e.g., physical and mental risks at work and personal life
**Using alternatives to full-time sick leave**	Prescribing sick leave if absence from work is required for recovery Using alternatives to full-time sick leave early on (e.g., compensatory work, part-time sick leave, work modifications)
**Advising patients with pain**	Enhancing patients’ understanding of pain and its mechanisms, treatment options, and the benefits of remaining active and staying at work

Twelve themes were retrospectively described by the implementers as barriers to the key behaviours during the launch of the guidelines. Table 2 presents the themes classified into COM-B components, and applied means (intervention functions) for addressing the barriers. It also shows whether the barriers were regarded by target physicians as currently influencing their behaviour. Detailed presentations of identified influences and applied means with sample quotes are provided in Additional files 2 and 4.

**Table 2:** Barriers to key guidelines-related behaviours and applied intervention functions to address the barriers

Barriers to key behaviours, identified by implementers, during the initiation of the guideline implementation intervention	COM-B component	Intervention functions applied to address the identified barriers	Identified as a barrier by some target physicians in 2020
Lack of knowledge and understanding of: • pain and treatment of pain, especially chronic pain • how to assess work disability and need for sick leave • consequences of (prolonging) sick leaves for employees and employers	Psychological Capability	Education	Yes Yes
Forgetting to take into account all relevant factors, when making decisions about pain treatment, work disability, need for sick leave, and alternatives for full-time sick leave	Psychological Capability	Enablement	
Routinized practice, based on outdated knowledge learned long ago; without an awareness of the need for change	Psychological Capability	Enablement	
Lack of time to engage in recommended behaviours, experienced especially by OHS general practitioners.	Opportunity: Physical environment	Environmental restructuring	Yes
Scarcity of admission hours to occupational physicians	Opportunity: Physical environment	None	Yes
Scarcity of non-pharmacological pain treatment tools in the OHS	Opportunity: Physical environment	Environmental restructuring	Yes
Doubts about personal capability to handle difficult situations with patients	Reflective Motivation	Education	
Perception of guidelines as restricting professional autonomy	Reflective Motivation	Persuasion	
Anticipation of negative consequences to oneself for applying the guidelines as recommended	Reflective Motivation	Persuasion	Yes
Intention not to learn new guidelines-related behaviours if learning seems burdensome	Reflective Motivation	Persuasion	

Five barriers to key behaviours were mapped to Psychological capability. These have been targeted mainly by intervention functions Education and Enablement. According to the implementers, many physicians lacked knowledge and understanding related to the key behaviours, resulting in undesirable behaviours: focus on precise diagnosing at the expense of finding out comprehensively factors related to pain; unnecessary referrals to imaging and specialists (often keeping patients on full-time sick leave waiting for further examinations); low use or late initiation of alternatives to full-time sick leave; and inadequate advising of patients with pain. According to the implementers, physicians’ lack of knowledge had been addressed by introducing the guidelines, offering short educational sessions, an e-learning course and some coaching sessions led by a pain specialist. The OHS management have also given personal guidance to physicians who are noticed by the radiological nurses to make unnecessary referrals to imaging.

Implementers also assumed that, due to haste at appointments, all physicians might not remember to consider all relevant factors when making decisions concerning pain treatment and sick leaves. More experienced physicians were also seen susceptible to routinized behaviour, based on outdated knowledge learned long ago. To address these barriers, physicians were instructed to keep printed guidelines at practice table and use the included check-lists as reminders to consider all relevant factors and to serve as means of interrupting routinized behaviour. Physicians were also provided with a questionnaire to be filled with the patients in order to notice the threat of chronic pain.

Three barriers to key behaviours were mapped to Opportunity (Physical environment). These have been targeted mainly by intervention function Environmental restructuring. Implementers assumed that general practitioners did not always have time to engage in all guidelines-consistent behaviours. In addition, scarcity of appointment hours to occupational physicians was thought to delay the recommended care of patients with chronic pain. Implementers also described insufficient non-pharmacological pain treatment, partly due to the scarcity of these means in the OHS.

According to the implementers, pain-groups were set up for patients with chronic pain. Occupational health nurses were educated to take bigger role in non-pharmacological pain treatment. This was also thought to provide physicians with more time to focus on their special role, e.g., medical treatment, assessing work disability and need for sick leave. Implementers expressed no specific means of addressing the assumed lack of admission hours to occupational physicians.

Four barriers to key behaviours were mapped to Reflective motivation. These have been targeted mainly by intervention functions Education and Persuasion. According to the implementers, physicians have low tendency to please patients but might give in for unfounded requests for sick leave if having doubts about their capability to handle difficult situations with patients. Implementers expected that key messages included in the guidelines would provide physicians with needed knowledge and encourage negotiations with all patients. Implementers anticipated that despite physicians’ general appreciation of evidence-based guidelines they might not follow the OHS guidelines if these would be perceived as restricting professional autonomy or bring about negative consequences to oneself (e.g., burdensome writing of expert statements for the application of part-time sick leave). In addition, some physicians might make a conscious decision to dismiss the new guidelines, if learning new behaviours would seem arduous. According to the implementers, educational sessions and monthly staff meetings have been used to deliver information about the expected and observed benefits of engaging in recommended behaviours. Physicians have also been partly involved in preparing the guidelines and later in discussing solutions to unexpected sick leave trends.

Target physicians’ perceptions of guidelines adherence, the intervention received, and current influences of recommended behaviours.

Table 2 shows which of the barriers to key behaviours, identified by the implementers during the launch of the intervention, were perceived as current influences of behaviour by the target physicians. Detailed presentation of physicians’ perceptions and experiences of guidelines and the intervention received, with sample quotes, are provided in Additional files 3 and 4.

All interviewed physicians were not familiar with the guidelines, as they had started working at the OHS after the launch of the intervention. However, when browsing through the guidelines during the interviews, even they estimated practising mostly as recommended. Physicians reported keeping absence from work as short as possible and using actively alternatives to full-time sick leave. Especially occupational physicians underlined the importance of finding out comprehensively factors related to pain and finding solutions in close co-operation with supervisors at the workplaces. Various examples of advising patients were described in the interviews.

Several factors were identified from the physicians’ interviews as facilitators of recommended behaviours. Factors pertaining to Psychological capability included sufficient knowledge and understanding, good interpersonal and negotiation skills, remembering to consider relevant factors when making decisions, and being accustomed to evaluating the appropriateness of one’s practice. However, some physicians with less working experience perceived scarcity of knowledge for diagnosing pain and/or assessing work disability and need for sick leave as hindering the expected behaviours.

With regard to Physical opportunity, some physicians mentioned barriers to key behaviours: scarcity of means of non-pharmacological treatment at the OHS; lack of time to examine the patient’s situation comprehensively and/or advise the patient during the appointment; and lack of admission hours to occupational physicians. However, others described having sufficient resources and mentioned these factors as facilitators of expected behaviours.

In relation to Social opportunities, physicians appreciated multi-professional co-operation and social support received from OHS seniors and colleagues. Many patients’ and supervisors’ positive attitude to suggested alternatives to sick leave was also seen to facilitate adherence to the guidelines. According to the physicians, specific professional and cultural norms enhancing guidelines-related behaviours prevailed in the OHS, but they had divergent opinions of the level of commitment to the norms among all colleagues.

With regard to Reflective motivation, physicians thought highly of the objectives of the guidelines, i.e., enhancing staying at work/early return to work. They perceived pain management and advising patients as important parts of professional role. The guidelines were regarded as enabling much professional freedom, and, according to the physicians, individuals’ guidelines-related behaviours are not monitored in the OHS. Although physicians believed that engaging in recommended behaviours would bring positive consequences to employees and the employer, some physicians anticipated negative consequences to oneself from e.g., using the printed guidelines as recommended with patients (patients might doubt their clinical ability). Physicians mentioned being encouraged to adhere to the guidelines through the enthusiasm shown by the implementers and seeing the trends of pain-related sick leaves in monthly staff meetings.

Especially physicians with less working experience reported having made explicit behaviour changes during the intervention: prescribing shorter sick leaves; using recommended pain treatment; encouraging patients more systematically to activity and staying at work; and referring patients with the threat of chronic pain more rapidly to occupational physicians. According to the physicians, behaviour changes were brought about by new knowledge received from the OHS educational sessions and especially from the guidelines. Knowledge and understanding gained at work through increasing work experience and from consultations with seniors and specialists were mentioned as influencing their behaviour. Other sources of knowledge were specialist training for occupational physicians, and courses, guidelines and medias provided for physicians by various Finnish organizations. In addition, the accumulation of working experience was considered important for behaviour change because it enhanced beliefs about one’s capability to handle challenging situations.

Some physicians reported using the guidelines check-lists as a reminder to recommended practice. In addition, the OHS electronic patient record system, introduced in 2018 and guiding physicians to take a stand on patient’s suitability to alternative work, was specifically mentioned as a reminder. Some physicians had used the guidelines check-lists as a means of assessing the appropriateness of one’s practice. Other means of self-assessment were feedback from patients and OHS seniors, and comparing one’s current behaviours to information received in educational sessions outside the OHS.

Description of trends of MSD-related sick leave days prescribed in the OHS before and after the launch of the guidelines.

**Fig. 1:**
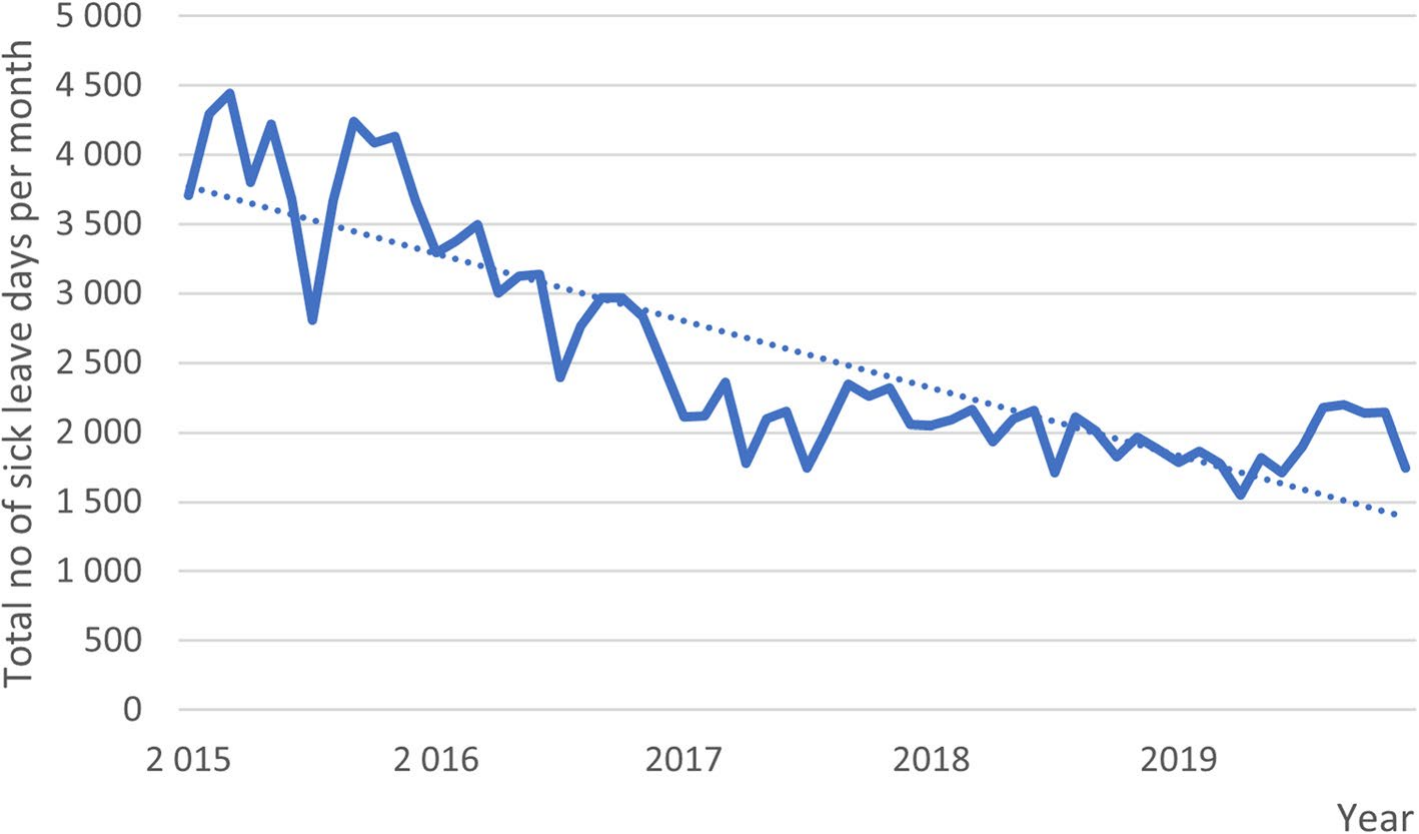
Total number of sick leave days prescribed at the OHS per month

**Fig. 2:**
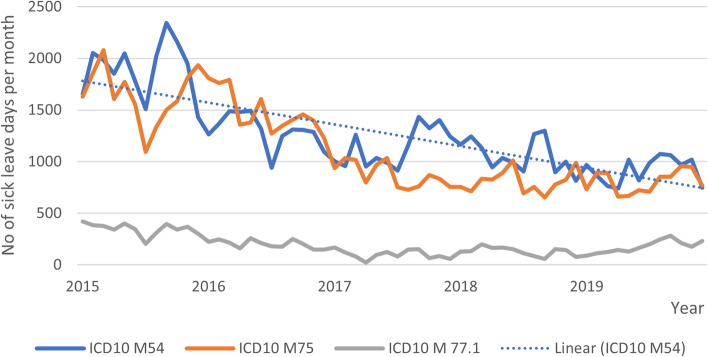
Total number of sick leave days prescribed at the OHS in three diagnostic categories (ICD10 M54, ICD10 M75, ICD10 M77.1)

The absolute total numbers of sick leave days in back pain, shoulder pain and elbow pain prescribed at the OHS are illustrated per month. The three diagnostic categories (ICD10 M54, ICD10 M75, ICD10 M77.1) are combined in Fig. 1 and presented separately in Fig. 2.

The number of sick leave days per month varied from 2290 to 689 in back pain, from 2012 to 652 in shoulder pain and from 417 to 23 in elbow pain during the follow-up. Overall, the descending trend detected in sick leave days was particularly noticeable in the categories of back pain and shoulder pain. In 2015, before the intervention was launched, the categories of back pain and shoulder pain were the two main ICD-related categories, composing about 89% of all MSDs. The actual results of the analyses in the register data are presented elsewhere (34).

Interviewees’ perceptions of factors beyond physician behaviours influencing the number of MSD -related sick leave days.

Implementers attributed the reductions in MSD-related sick leaves largely to physicians’ engagement in guideline-consistent behaviours. Target physicians were more cautious in their evaluations. All interviewees (including occupational physiotherapists and an occupational health nurse) suggested several factors beyond physicians’ behaviours that may influence the number of MSD-related sick leave days (see Table 3). Detailed presentations of these factors, with sample quotes, are provided in Additional file 5.

**Table 3:** Factors beyond physicians’ behaviours influencing the number of MSD-related sick leave days

Factors	Themes of factors
**Treatment provided by the OHS multi-prosessional personnel (in addition to physicians)**	Patients with minor symptoms are guided to effective self-care by nurses during the assessment of need for treatment ^a^ Occupational health nurses provide non-pharmacological care for patients with chronic pain^a^ Physiotherapists provide effective pain treatment and guide patients in self-care^a^ Occupational health psychologists support patients with chronic pain^a^ Full-time physiatrist and psychiatrist serve patients with chronic pain^a^ Shortage of appointment hours for occupational health psychologists and physiotherapists^b^
**Policy, structures and processes in the OHS**	OHS revenue logic allows professionals to focus on the prevention of work disability^a^ Fairly well-operating assessment of need for treatment and referral to appropriate professionals^a^ Well-functioning distribution of work among general practitioners and occupational physicians^a^ Self referral to physiotherapists^a^ Sick leave trends are systematically monitored in the OHS and possible setbacks are discussed in staff meetings^a^ OHS actively informs workplaces about new policy and practices concerning e.g., sick leave prescribing^a^ OHS actively supports workplaces in using alternatives to sick leave^a^
**Municipal client organization**	Client organization is committed to enhancing the use of alternatives to full-time sick leaves^a^ Client organization provides employees with services for enhancing health and preventing work disability, e.g., groups supporting healthy behaviours^a^ Alternatives to full-time sick leave (e.g., work modifications) are difficult to carry out at some duties or workplaces^b^
**Employees of the client organization**	Positive cultural change is noticed in most employees’ attitudes concerning absence from work^a^ Employees’ willingness to avoid absence from work is influenced by current labour market situation and the level of unemployment benefits^a,b^ Many employees engage in unhealthy behaviours, which affects work ability in the long run^b^
**Structures and processes in primary and special health care, and social insurance**	Sick leaves are easily prolonging due to insufficient treatment of pain in primary and special health care^b^ Long waiting lists to special health care (e.g., in need for operations) extend sick leaves^b^ Social insurance regulations hinder the use of part-time sick leave^b^

^a^Factors are perceived to decrease sick leave days; ^b^Factors are perceived to increase sick leave days

According to the interviewees, the OHS revenue logic (no expectations to produce returns to owners) and structures and fairly well-operating processes ensure that the multi-professional personnel is able to focus on preventing pain-related work disability and controlling prolonging sick leaves. Of specific actions, they emphasized the role of direct access to physiotherapists as an important organizational change which had reduced sick leaves. The commitment of the client organization to supporting employees’ work ability and staying at work was also seen essential. Prior OHS media campaign targeted to the employees and supervisors was estimated to contribute to their mainly positive attitudes towards avoiding absence from work and using alternatives to full-time sick leaves. However, the interviewees also named factors increasing MSD-related sick leaves, including shortage of appointment hours for professionals during the time of the interviews; difficulty of carrying out alternatives to full-time sick leave at some duties or workplaces; employees’ unhealthy lifestyles; insufficient treatment of pain in primary and special health care; long waiting lists to special health care; and social insurance regulations delaying the use of part-time sick leave.

## Discussion

The OHS guidelines, launched in 2016, were part of a multi-component project aiming to prevent and reduce MSD-related work disability and sick leaves in a large municipal organization. The guidelines aimed to decrease between-physician variation in sick leave prescribing, increase the use of alternatives to full-time sick leave early on, enhance pain management, and influence patients’ pain-related beliefs and attitudes. According to the intervention implementers, the aims had been achieved well. Target physicians, in turn, reported adhering largely to the guidelines and described corresponding behaviours as well as various facilitators of these behaviours. We also identified factors that may have contributed to the observed trends in pain-related sick leaves beyond physicians’ behaviours.

Six of the twelve barriers to key behaviours, identified by the implementers during the initiation of the intervention (e.g. lack of knowledge on the consequences of prolonging sick leaves or doubts about personal capability to handle difficult situations with patients) were not reported as current hindrances to practice by any of the interviewed target physicians. Instead, they described several facilitators of recommended behaviours. It seems that these barriers had been overcome by means applied in the intervention or by external means. However, as we relied on physicians’ self-assessment, it is possible that some barriers identified by the implementers (e.g., routinized practice based on outdated knowledge or forgetting to take account of all relevant factors when making decisions about pain management or sick leave) still prevailed but were not recognized or reported by all interviewed physicians. Some physicians, however, described also means to enhance remembering and reflecting on one’s practice.

Target physicians’ reports of explicit behaviour changes suggest that the intervention achieved its most effects by enhancing physicians’ Psychological capability to engage in recommended behaviours via the intervention functions Education and Enablement. Especially physicians with less working experience described having gained new knowledge and understanding about essential themes from the OHS guidelines. The role of the OHS’s own education may have been larger during the initiation of the intervention. For example, referrals to imaging had been noticed to reduce markedly after the education. However, the majority of the interviewed physicians had started working in the OHS after this education. Physicians’ knowledge has been increased also through learning at work when engaging in new tasks and by other pathways beyond the OHS intervention.

As intended by the implementers, the guidelines were used at least by some interviewees as a reminder of recommended practice and/or a means of self-assessment, i.e., comparing current practice against the guidelines. Physicians appreciated the electronic patient record system, separate from the initial intervention, for guiding them to take a stand on patient’s suitability to alternative work when completing the sick leave prescription.

Physicians identified several facilitators of guidelines-consistent behaviours which were mapped to Reflective motivation and Social opportunity, but for the most part they were not directly related to the intervention received. For example, some physicians explained that they had chosen to work in this OHS because it enabled practicing according to one’s professional goals (preventing work disability, enhancing staying at work/early return to work). Possibilities to multi-professional co-operation and a culture of collegial social support were reported to have prevailed in the OHS even before the intervention. Long-time active collaboration between the OHS and workplaces was seen to result in patients’ and supervisors’ mostly positive attitudes towards the alternatives to sick leave and, thus, facilitate negotiations at the physician’s office. Media campaign targeted to workplaces, as part of the broader OHS development project, was estimated to have a separate positive effect.

Six of the twelve barriers identified initially by the implementers were reported as current hindrances to practice by some target physicians also. Some physicians, especially those with less experience in occupational health care and/or medical practice in general mentioned lack of knowledge. It seems that the intervention has not addressed sufficiently barriers pertaining especially to Physical opportunity. Lack of time to engage in recommended behaviours was experienced especially by general practitioners. Increasing occupational health nurses’ role in chronic pain management may have been insufficient because general practitioners see mostly patients with acute pain. Scarcity of admission hours to occupational physicians concerned some but not all OHS teams. Some physicians recognized non-pharmacological pain treatment tools available in the OHS while others described a shortage of these means, which may be due to lack of information. These results underline the importance of taking into account the needs of different subgroups when implementing an intervention [26, 27]. In addition, process evaluations of ongoing interventions would bring out needs for refinement [19].

We obtained and illustrated data on MSD-related sick leave days prescribed in the OHS before and after the launch of the guidelines using a five-year follow-up period. A descending trend was detected in the number of sick leave days per month especially in low back pain and shoulder pain. However, the effectiveness of the intervention on employees’ sickness absence cannot be evaluated in this study due to the absence of a control group. The results of this analysis are reported elsewhere [34]. However, it seems evident that considerable changes took place during the time period.

Interviewed OHS professionals estimated that the guidelines and related physicians’ behaviours had impacted the observed trend, but they also identified factors beyond physicians’ behaviours, which may have reduced the number of sick leave days: direct access to physiotherapists, good treatment provided by all OHS professionals and enabled by the OHS policy and fairly well-functioning structures and processes, commitment of the client organization to supporting its employees’ work ability, and a cultural change in employees’ attitudes towards absence from work. These factors may also indirectly facilitate physicians’ guidelines-consistent behaviours.

Interviewees also described factors beyond physicians’ behaviours as increasing the number of MSD-related sick leave days, such as structures and processes in primary and special health care, and social insurance. Alternatives to full-time sick leaves are also difficult to carry out in some duties or workplaces, e.g. in heavy manual work.

According to previous studies, primary care physicians found challenging to assess and negotiate the need of sick leave with patients with MSD [3, 4, 35], and sick-listing in general [36–39]. Physicians reported of personal lack of knowledge, skills and motivation but also of patients and supervisors who were reluctant to suggested alternatives to sick leave. Challenges to recommended sick-listing pertained also to health care organization, e.g., inadequate leadership, insufficient incentives and support for handling sickness certification. In addition, physicians reported of barriers related to societal level, such as long waiting time in health care and inadequate cooperation between different stakeholders in health care and social insurance. Challenges experienced by physicians may result in prescribing unnecessarily long sick leaves [2, 38].

Interventions to promote physicians’ recommended sick leave prescribing may be successful, e.g. encourage them to discuss work with patients or use part-time sickness absence more often [3, 40, 41], but a physician is only one actor in a network of several stakeholders. Targeting physicians only does not, for example, influence the workplaces’ policies for using alternatives to sick leaves. Our results suggest that the OHS has successfully addressed many of the challenges identified in previous studies. Physicians’ capabilities and motivation were explicitly addressed in the intervention but it seems that the OHS provided most interviewed physicians with adequate physical and social environment also. Good long-standing collaboration with the workplaces facilitated engagement in recommended practice. However, also physicians in the present study described societal level barriers to decreasing the number of sick leave days. These factors are beyond the means of interventions implemented by single health care organizations.

Prior studies [17, 42] have presented contradictory results on whether application of various means versus single or few means during an intervention (e.g., dissemination of guidelines, educational meetings, audit and feedback) increased desired physicians’ pain management behaviours. However, instead of trying out different intervention means only, increasing emphasis is now placed on using appropriate theories when developing behaviour change interventions [28, 43]. According to the intervention head designer, the OHS intervention was planned without an explicit behavioural theory but it drew on research on pain management and sickness absence prevention, domestic and foreign pain-related guidelines, and lessons learned from prior interventions among physicians. The designer’s own experience in the field informed which physician behaviours should be targeted, and which intervention strategies are likely to be effective and feasible, given the available resources and the likelihood of acceptance to the physicians. The implementers knew well the OHS staff at that time and the challenges in preventing and reducing MSD-related work disability in this context. Prior studies [13–16] have also highlighted the importance of identifying and targeting context-specific barriers to desired behaviours.

The declining trend of MSD-related sick leave days prescribed in the OHS levelled off somewhat during the five-year follow-up period. The implementers had noticed variance in the trends of sick leave days especially during summer months suggesting that locum physicians are not introduced sufficiently to the guidelines. In addition, we identified some changes in the intervention process during the follow-up years. Physicians new to the OHS were supposed to be introduced to the guidelines, but not all of our interviewees were familiar with them. Some of the initial intervention components are not in use anymore, i.e., education provided by the OHS, the pain questionnaire to assist physicians in noticing patients with threat of chronic pain, and the pain groups for patients. It seems that no compensatory means have been provided to the physicians. Both the implementers and target physicians considered the current lack of pain-related education as a hindrance to supporting the recommended behaviours.

We have used social and health psychological knowledge for conducting this intervention evaluation, but the results can also be discussed from the perspective of medical sociology. Occupational health care generally operates within biomedical paradigm. Biomedicine is often criticized for neglecting to give proper attention to the socio-cultural dynamics of pain care [44], and for failing to address the extent to which the pain sensation takes place as a product of interaction between neurophysiological processes, social contexts (e.g., working life) and cultural meaning [45–47]. Generally speaking, the OHS guidelines, aiming at better self-treatment of pain, reduction of medically certified absences, and improvement of physicians’ capability to deal with pain patients, can be viewed as an intention to de-medicalize pain care within a medical institution. The guidelines implementation can be represented as an interdisciplinary approach that intends to make the lived experience of human suffering an object of caring concern [46], providing social and psychological non-pharmacological tools for pain patients. Our results suggest that this type of approach with de-medicalization flavor may be effective but as the OHS pain patients were not under the scope of this study, we are not able to analyze how the changes in OHS were viewed among them.

The strength of this study is the use of descriptive quantitative data and qualitative data. Interviews with the OHS professionals allowed us to investigate possible factors influencing the observed sick leave trends. We used three different interview data sources, i.e., the implementers, target physicians and other OHS professionals, adding to the comprehensiveness of the data. Interviews with the implementers enabled us to characterize the OHS guidelines implementation intervention and to explore how it may have produced its effects. Implementers provided necessary information about the context and content of the intervention and allowed us to identify which of the outcomes, reported by the target physicians, could be attributed to the intervention and which to factors external to the intervention. For example, an e-learning course was a planned intervention means but rarely utilized by the target physicians. Instead, they reported gaining new knowledge from different sources outside the OHS. Implementers’ descriptions of the rationale behind the intervention allowed us to identify what physician behaviours they were trying to change, which barriers to the key behaviours they had identified and whether the barriers were targeted by theoretically appropriate intervention functions [26, 27]. It seems that key behaviours and their determinants had been explored and that there was theoretical coherence between identified barriers and means applied to address these barriers.

This is the first study, to our knowledge, to use the BCW and included COM-B model to evaluate a behaviour change intervention targeted to physicians in occupational health context. A theory-informed evaluation enabled us to explore how the intervention may have produced its effects. This examination also allowed the identification of current barriers to recommended practice which are not adequately addressed, suggesting needs for refinement of the ongoing intervention.

The COM-B model underlines the inter-connectedness of the three components [26]. Our data shows, for example, that occupational physicians used the printed guidelines check-lists with patients as recommended, when they had relevant knowledge and adequate time resources at the practice, but also believed that this behaviour benefits both patient and physician. On the other hand, some physicians were not aware of the check-lists (lacked knowledge), and the recommended behaviour did not occur despite adequate time resources and a motivation to serve the patients well. As for the general practitioners, they sometimes dismissed advising patients in time pressure even when they regarded this task as an important part of professional role and knew what to say to the patients.

The study has some limitations. Only approximately half of the invited OHS physicians volunteered to participate in the study. The appearance of COVID-19 pandemic increased physicians’ work strain and diminished willingness to contribute. It is difficult to recruit physicians for studies, and so this sample is quite good considering this hard-to-reach population. We collected an extensive amount of data on the topic and similar views and opinions started to appear during the course of the interviews (data saturation). The results may be biased to overestimate how the guidelines are implemented in the OHS. The interviewees may be likely to adhere to the guidelines more than others, or may represent a special subgroup of the staff with regard to some other issue. Thus, important barriers to guidelines-consistent behaviours may have been left unidentified in this study. However, participants varied with regard to their awareness of and ways of using the guidelines.

We had no data on physicians’ actual guidelines-related behaviours but relied on their self-assessments and descriptions of behaviours. In addition, the long intervention period is likely to affect the accuracy of memories of those who designed the intervention and those who have received it. Several intervention means may have been used but not recalled anymore during the interviews. We did not investigate factors influencing the delivery of the intervention itself. It is recommended that intervention evaluations start already during the launch of the intervention [19].

We identified specific behaviours (e.g. diagnosing and treating pain, assessing work disability) from the bundle of guidelines-related behaviours but enquired the interviewees’ perceptions of barriers and facilitators more generally. Influences of key behaviours may be different depending on the behaviour in question [6, 33, 38]. Furthermore, each of the key behaviours may consist of several sub-behaviours. A more fine-grained questioning would have required longer interviews but might have revealed relevant additional influences.

This study was conducted in Finland with a special occupational health care system. Industrialised countries differ considerably with regard to the content and coverage, organization and staffing of OHS [48]. Finnish occupational physicians have good possibilities to influence the number of sick leaves. In Finland, all employers must provide preventive occupational health care for their employees, and most employers also provide medical care at general practitioner level [49]. In 2015, OHS covered 84% of the employed workforce and 96% of wage earners in Finland, while, for example in Sweden, about 66% of the working force had access to OHS [49, 50]. In Sweden, occupational physicians prescribe small proportion of all sickness certificates, compared to occupational physicians, for example, in Finland and the Netherlands [50, 51].

Furthermore, the results come from a single OHS which, contrary to many corresponding Finnish organizations, serves only one client organization and has a unique revenue logic. Therefore, we are cautious to generalize the results to other contexts. Factors influencing physicians’ guidelines-related behaviours may be context-specific [6, 16]. However, we believe that the utilization of the BCW might be helpful in identifying barriers and facilitators of practice in a variety of jurisdictions and contexts. Further theory-informed research on the influences of behaviour is needed in other health care settings for more specific recommendations for intervention development.

## Conclusions

Evaluation of the OHS guidelines implementation intervention suggests that its aims were achieved well. Target physicians reported adhering to the guidelines and described corresponding behaviours as well as various facilitators of these behaviours. A descending trend was detected in the number of sick leave days per month prescribed in the OHS after the launch of the guidelines. Physicians’ explicit behaviour changes were attributed mainly to increased knowledge, disseminated partly through the intervention means. The study underlines the importance of supporting culture and policies, and structures and processes of the health care organization itself but also of well-functioning co-operation with key stakeholders in question, e.g., the employees and supervisors.

## Supplementary Information

**Additional file 1.** Interview protocols. The file includes interview protocols for different parties (intervention implementers, target physicians, other occupational health care professionals).

**Additional file 2.** Summary of facilitators of and barriers to guidelines-related behaviours, as perceived by the implementers. The file includes a summary of facilitators of and barriers to guidelines-related behaviours as perceived by the implementers, classified into COM-B components, with sample quotes.

**Additional file 3.** Summary of facilitators of and barriers to guidelines-related behaviours, as perceived by the target physicians. The file includes a summary of facilitators of and barriers to guidelines-related behaviours as perceived by the target physicians, classified into COM-B components, with sample quotes.

**Additional file 4.** Summary of intervention means applied by the implementers and experienced by the target physicians. The file includes a summary of intervention means applied by the implementers to target the identified barriers to guideline-related behaviours and experienced by the target physicians, with sample quotes.

**Additional file 5.** Summary of factors, beyond physician behaviours, influencing the number of MSD-related sick leave days. The file includes a summary of factors, in addition to physician behaviours, influencing the number of MSD-related sick leave days, as perceived by the OHS professionals, with sample quotes.

## Data Availability

The datasets generated and analysed during the current study are not publicly available according to the informed consent signed by the participants, but are available from the corresponding author on reasonable request.
