# Supplementary material for: Evaluation of a guidelines implementation intervention to reduce work disability and sick leaves related to chronic musculoskeletal pain: a theory-informed qualitative study in occupational health care
Source: BMC Musculoskelet Disord. 2022 Mar 22;23:272. doi: 10.1186/s12891-022-05234-8 (PMC8938719; doi:10.1186/s12891-022-05234-8)
Supplement: Supplementary file 1 — Additional file 1. Interview protocols. The file includes interview protocols for different parties (intervention implementers, target physicians, other occupational health care professionals). [file 12891_2022_5234_MOESM1_ESM.pdf]

Additional file 1. Interview protocols

In the beginning of the interviews, participants were asked to complete a background information form, including following questions:

- How long have you been practising as a general physician/occupational physician/occupational physiotherapist/occupational health nurse?
- How long have you been practising in this OHS?
- How long have you been aware of the OHS guidelines?
- Approximately, what proportion of your patients have musculoskeletal disorders?
- Approximately, how many MSD patients do you encounter in a typical working day in your practice? With how many of them you apply the OHS guidelines?

Interview protocol for interviews with the implementers

Interview topics:

- Background and aims of the OHS guidelines:
    - when and how the guidelines were formulated;
    - to whom the guidelines were targeted;
    - what problems had been identified concerning pain management and the assessment of work disability and need for sick leave among OHS professionals.
- Key guidelines-related behaviours and factors influencing these behaviors:
    - what physician behaviours were central in achieving the aims of the guidelines;
    - which factors facilitated and hindered those behaviours.
- Guidelines implementation intervention:
    - how the intervention was designed;
    - which intervention means were used and why;
    - which factors have facilitated and hindered the implementation.
- Effects of the guidelines implementation intervention on physicians' behaviour:
    - how target physicians have responded to the guidelines;
    - how the intervention effects have been measured;
    - what intervention effects have been identified;
    - what other factors (beyond the guidelines implementation intervention) may have influenced physicians' (possible) behaviour changes.
- Effects of the OHS guidelines on MSD-related sick leave days prescribed in the OHS:
    - how the number of sick leave days have varied before and after the launch of the guidelines;
    - which factors, in addition to physician behaviours, influence the number of sick leave days.

Interview protocol for interviews with the target physicians

Participants in the physician interviews were first shown one printed guidelines (low back pain).

Interview topics:

- Participants' awareness of the OHS guidelines:
    - whether they are aware of the guidelines;
    - when and how they had been introduced to the guidelines;
    - if not aware, participants are asked to browse through the printed guidelines concerning low back pain and estimate to what extent they practice according to the guidelines.
- Application of the guidelines:
    - how many patients with low back pain, shoulder pain or elbow pain they encounter on a typical working day;
    - how they apply the guidelines with these patients;
    - whether they use the printed guidelines to advise a patient and/or complete the guidelines check-list with a patient;
    - if they are not applying the guidelines with a patient, why not.
- Perceptions of the guidelines:
    - whether they perceive the OHS guidelines as useful, and why (not);
    - whether (and in what way) physicians benefit from the guidelines.
- Possible behaviour changes induced by the guidelines:

    - whether they have changed their practice after becoming aware of the guidelines, and if so, in what way;
    - which other factors have possibly induced changes in their guidelines-related behaviours;
    - if no behaviour changes have been made, why not.
- Capability to practice according to the guidelines:
    - what kind of training, beyond basic medical education, they have received concerning guidelines-related behaviours;
    - whether they recognize needs for more knowledge or skills;
    - whether remembering to practice according to the guidelines is an issue to them.
- Motivation to practice according to the guidelines:
    - the importance of pain management and reducing MSD-related sick leaves in their professional role;
    - to what extent the aims of the guidelines are priorities for them;
    - what benefits and disadvantages (and to whom) they perceive of practising according to the guidelines;
    - how physicians' adherence to the guidelines is monitored in the OHS;
    - whether they have experienced the negotiations about pain management and/or sick leave challenging with some patients;
    - whether any specific emotions are attached to seeing patients with MSD and/or applying the guidelines.
- Opportunities to practice according to the guidelines:
    - which aspects of the physical environment influence whether they practice according to the guidelines;
    - how other OHS professionals influence whether they engage in recommended behaviours;
    - how the OHS as an organization influences their adherence to the guidelines;
    - how the patients and supervisors in the workplaces influence whether they practice according to the guidelines.
- Effects of the OHS guidelines on MSD-related sick leave days prescribed in the OHS:
    - how has the number of sick leave days varied before and after the launch of the guidelines;
    - which factors, in addition to physician behaviours, influence the number of sick leave days.

Interview protocol for interviews with other OHH professionals (occupational physioterapists and nurses)

Interview topics:

- Participants' roles and duties in pain management in the OHS
- Multiprofessional co-operation in the OHS
- Barriers to and facilitators of recommended pain management in the OHS
- Participants' awareness and possible application of the OHS guidelines
- Participants' perceptions of adherence to the guidelines among OHS staff
- Participants' perceptions and possible experiences of the guidelines implementation intervention
- Participants' perceptions of factors influencing the number of MSD-related sick leave days
