# Supplementary material for: Evaluation of a guidelines implementation intervention to reduce work disability and sick leaves related to chronic musculoskeletal pain: a theory-informed qualitative study in occupational health care
Source: BMC Musculoskelet Disord. 2022 Mar 22;23:272. doi: 10.1186/s12891-022-05234-8 (PMC8938719; doi:10.1186/s12891-022-05234-8)
Supplement: Supplementary file 2 — Additional file 2. Summary of facilitators of and barriers to guidelines-related behaviours, as perceived by the implementers. The file includes a summary of facilitators of and barriers to guidelines-related behaviours as perceived by the implementers, classified into COM-B components, with sample quotes. [file 12891_2022_5234_MOESM2_ESM.pdf]

Additional file 2. Summary of facilitators of and barriers to guidelines-related behaviors as perceived by the implementers, classified into COM-B components, with sample quotes

Imp1 = Implementers' interview 1; Imp2 = Implementers' interview 2

Reference: Michie S, van Stralen MM, West R. The behaviour change wheel: A new method for characterising and designing behaviour change interventions. *Implement Sci* 2011;6:42.

Psychological capability

An individual's ability to engage in a behavior in terms of knowing what to do, how to do it and why it is worth doing; and having the psychological skills, strength or stamina to engage in the necessary mental processes (Michie et al. 2011).

Facilitators (F) and barriers (B) identified by the implementers

- B: Many physicians lacked knowledge and understanding of pain, and diagnosing and treating (chronic) pain: *"In Finland, we have quite an inadequate training for the treatment of pain. The approach has been very biomechanic. Physicians are taught to diagnose pain and look for its cause, and a lot of time is spent there. The opinions of physiatrists and orthopedists are overvalued. A holistic approach is missing from the treatment of pain. The focus is placed on correcting the ergonomics only. Too many restrictions are imposed too lightly and too early."* (Imp1)
- B: Many physicians lacked knowledge of how to assess work disability and the need for sick leave*: "Everybody has trusted the gut feeling. The results of a thesis showed that there are up to eight-fold differences between the length of a sick leave prescribed by different physicians for the same malady."* (Imp1)
- B: Many physicians lacked knowledge of the negative consequences of (prolonging) sick leaves for employees and employers: *"Physicians very often start unnoticed a process of a prolonged sickness absence, without understanding the hazards of such a process. One does not understand that prescribing a sick leave is one of the most expensive medical interventions available to a phycisian."* (Imp1)
- B: Especially physicians with less working experience were prone to forget to take into account all relevant factors, when making decisions about pain treatment, work disability and need for sick leave: *"For example in elbow pain, inexperienced physicians could refer the patient right away to physical therapy and forget to find out the origin of the pain, whether it be the work or leisure or both."* (Imp2)
- B: Especially experienced physicians were susceptible to routinized practice, based on outdated knowledge learned long ago: "*Many colleagues think that sick leave is something the patiens are looking for, and this automatic process of sick leave prescribing keeps rolling on. When referring a patient to a specialist, it is also quite common to keep the patient on sick leave for the entire period of queing to the specialists' surgery"*. (Imp1)

Physical opportunity

Opportunity afforded by the physical enviroment, such as time, finances, locations, materials, cues (Michie et al. 2011)

Facilitators (F) and barriers (B) identified by the implementers

- B: Especially general practitioners lacked time to engage in all recommended behaviors: *"General practitioners have tight schedules at the practice, and one does not have enough time to consider alternatives to sick leave. One has to balance between examining and informing the patient. There is not enough time for everything."* (Imp1)

- B: Physicians seldom used non-pharmacological tools in treatment of (chronic) pain due to the scarcity of these tools in the OHS: *"Chronic pain is a challenge for the health care professionals, especially when all the treats for acute pain have been tried out and work disability is beginning to show".* (Imp2)
- B: Lack of admission hours to occupational physicians resulted in prescription of overly long sick leaves: *"General practitioners have only a short time for each patient. It may take from six to eight weeks before a patient is assigned to a team of occupational specialists, who may have the time to properly treat the patient. There is a general lack of appointment hours for our own occupational phycisians."* (Imp2)

Social opportunity

Opportunity afforded by the social and cultural milieu, such as social structures, social support, role models, cultural norms and linguistic/conceptual structures that dictates the way that we think about things (Michie et al. 2011).

Facilitators (F) and barriers (B) identified by the implementers

- F: Majority of patients responded positively to non-pharmaceutical treatment and alternatives to full-time sick leave: *"People mostly feel positive about non-pharmacological treatments without sick leave. In my experience, most patients in pain are not looking for sick leave but want to continue at work and seek information about the cause and eventual treatment of their pain."* (Imp1)

Reflective motivation

Reflective processes involving intentions, plans and evaluations (Michie et al. 2011)

Facilitators (F) and barriers (B) identified by the implementers

- F: Occupational physicians perceived active co-operation with employers as an important part of their professional role: *"In the OHS, occupational physicians are taught from day one that it is their duty to be active and contact the workplaces. Things don't work out if one acts alone only and keeps prescribing sick leave. One has to communicate."* (Imp1)
- F/B: Physicians were willing to adhere to evidence-based guidelines / Physicians might perceive the OHS diagnosis-specific guidelines as restricting professional autonomy: *"The Social Insurance Institution has studied the willingnes of physicians to receive these recommendations and it is found to be very high. Physicians are very happy about this kind of guidelines and recommendations."* (Imp1) /*"It is known that physicians do not pay much attention to recommendations. The management has to be really assertive or everyone will follow their own ways regardless of the recommendations"*. (Imp2)
- B: Physicians might doubt their personal capability to act as recommended, e.g., to negotiate the need for sick leave with a patient in case of disagreement: *"Certainly, physicians wish to avoid conflicts with patients when discussing the need of a sick leave. They don't want to frustrate patients or make them angry in case of a denial of a sick leave."* (Imp1)
- B: Physicians might engage in non-recommended practice, e.g., prescribing full-time sick leaves instead of discussing alternatives, in order to avoid presumed negative consequences to oneself: *"You don't change your behaviour unless you really understand why you should run the extra mile to treat this patient, when you could spare the effort. You want to stick to your schedule, you might even be heading for the lunch or for the next patient. Should I just go ahead and print out the sick leave certificate or contact the patient's supervisor instead?"* (Imp1)
- B: Physicians might make a conscious decision not to change practice if learning new behaviours seems burdensome: *"Unlearning old practices is quite an effort. If most physicians working even in occupational health are staggering on the brink of exhaustion, starting the process might be too much. New tools must be really simple and easy to use, because any additional strain may overwhelm them"*. (Imp1)

Automatic motivation
Automatic processes involving emotional reactions, desires, impulses and inhibitions, habits (Michie et al. 2011).

Facilitators (F) and barriers (B) identified by the implementers

- F: OHS physicians did not have financial incentives to engage in non-recommended behaviors, e.g., maximizing the number of patients per day or referrals to imaging: *"Most physicians working in the private sector are self-employed. You get a much higher pay for receiving three patients rather than just one per hour. In this OHS all pysicians receive monthly salary."* (Imp1)
