# Supplementary material for: Evaluation of a guidelines implementation intervention to reduce work disability and sick leaves related to chronic musculoskeletal pain: a theory-informed qualitative study in occupational health care
Source: BMC Musculoskelet Disord. 2022 Mar 22;23:272. doi: 10.1186/s12891-022-05234-8 (PMC8938719; doi:10.1186/s12891-022-05234-8)
Supplement: Supplementary file 3 — Additional file 3. Summary of facilitators of and barriers to guidelines-related behaviours, as perceived by the target physicians. The file includes a summary of facilitators of and barriers to guidelines-related behaviours as perceived by the target physicians, classified into COM-B components, with sample quotes. [file 12891_2022_5234_MOESM3_ESM.pdf]

Additional file 3. Summary of facilitators of and barriers to guidelines-related behaviors as perceived by the target physicians, classified into COM-B components, with sample quotes

Phys1 = Physicians' interview 1 … Phys9 = Physicians' interview 9

Reference: Michie S, van Stralen MM, West R. The behaviour change wheel: A new method for characterising and designing behaviour change interventions. *Implement Sci* 2011;6:42.

Psychological capability

An individual's ability to engage in a behavior in terms of knowing what to do, how to do it and why it is worth doing; and having the psychological skills, strength or stamina to engage in the necessary mental processes (Michie et al. 2011).

Facilitators (F) and barriers (B) described by the target physicians

- F: Physicians regard the OHS guidelines as trustworthy: *"There has been a specialist and a working group writing them (the guidelines). That sounds good to me. I trust their judgement."* (Phys1)
- F/B: Physicians know how to diagnose pain / Some physicians find challenging to diagnose pain: *"The same complaints recur, it is the back pain and shoulder pain, so it becomes a routine. Even though I am not very experienced, examining back and shoulder is already going well."* (Phys3) / *"Patients with musculoskeletal disorders have been sort of bogeys to me as I have felt that I cannot treat them very well. One seldom comes up with a specific diagnosis for example for low back pain. Sometimes it is difficult to detect if there is something else behind the pain or why the situation is not getting better*." (Phys9).
- F/B: Physicians know how to treat pain comprehensively (taking into account different factors influencing pain) / Some physicians find treating patients with chronic pain challenging: *"It is not always back or shoulder pain only. Many kinds of strain are often involved and have an impact on well-being at work. Patients may be depressed or have symptoms of depression, not always diagnosed. In these cases, we often take good use of multiprofessional cooperation."* (Phys7) / *"Occupational physicians see more often patients with chronic pain than general practitioners do. There are more often problems that affect work. I am not yet very experienced with chronic pain cases. I find that I will have to learn and study more."* (Phys3)
- F: Physicians know that encouraging patients' activity is central in pain management: *"In my opinion, we have a very rational and modern approach to this (pain management). Activity is favored. I do not think that anyone just prescribes sick leave and tells the patient to lay themselves down."* (Phys6)
- F/B: Physicians know how to assess patients' work disability and need for sick leave / Some physicians find the assessing of work disability and need for sick leave challenging: *"When it is a matter of work-related musculoskeletal disorder, I discuss it with the patient. If it is unclear to me what are the patients' work tasks, I keep asking silly questions until I understand the nature of the work. It is easier for me to prescribe short sick leaves now when I understand the requirements in different work situations. On the other hand, I have also learned to prescribe longer sick leaves when necessary."* (Phys4) / *"It is sometimes difficult to know how the pain affects getting along at work and is there need for sick leave. There are so many different occupations and I do not know what kind of tasks each of them includes. I am still rather inexperienced as a physician."* (Phys3)
- F: Physicians know the alternatives to full-time sick leave and their benefits: "*I find using alternative work rational. For example, if one is able to work from home while waiting for a knee surgery, it is a win-win situation. Sickness absence does not prolong right away."* (Phys1)
- F: Physicians have interpersonal skills, e.g., know how to discuss and reach consensus with patients: *"In, let's say, 90 percent of cases we finally agree. Sometimes I find that a patient would not need sick leave, but he/she disagrees. Then, if the patient feels not being able to go to work, I usually prescribe a short sick leave for a few days, depending on the case."* (Phys3)

- F: Physicians remember to take into account relevant factors when making decisions about pain management, work disability, need for sick leave and alternatives for full-time sick leave: *”Of course, the topics included in the guidelines have to be discussed with the patients. I find that I have done that anyway, but maybe the guidelines serve as reminders.”* (Phys6)
- F: Physicians are accustomed to evaluating the appropriateness of their professional practice: *”When the patient is back for the control appointment, you can always evaluate whether the prior decision was a good one or not.”* (Phys6)

Physical opportunity
Opportunity afforded by the physical enviroment, such as time, finances, locations, materials, cues (Michie et al. 2011)

Facilitators (F) and barriers (B) described by the target physicians

- F/B: Physicians can use non-pharmacological tools for pain treatment in the OHS / There are not enough possibilities for non-pharmacological pain treatment: *”In the OHS we have a variety of means to help the patients. They can get an appointment to a physiotherapist rather quickly. They can also contact occupational physiotherapists who visit workplaces as well.”* (Phys9) / *“I still prescribe pain medications quite a lot, so it would be better to have more means for non-pharmacological pain treatment. As far as I know, the pain groups do not exist anymore.”* (Phys1)
- F/B: General physicians can refer patients rapidly to occupational physicians / Patients cannot be referred to occupational physicians rapidly enough: *”There is quite a lot of flexibility in the schedules of the occupational physicians. However, this might depend somewhat on the particular team.”* (Phys7) / *“Sometimes it is difficult to get a patient referred to occupational physicians. Their calendars may have been full for two months ahead.”* (Phys2)
- F/B: Occupational physicians have appointment hours to their own patients / Some occupational physicians do not have sufficiently appointment hours: *”If I have a patient with pain needing sick leave, I reserve an appointment for the last day of sick leave. I usually tell the patient, that if you then feel okay you can cancel the appointment and go to work. There are such free spaces in my calendar.”* (Phys8) / *“If my calendar is full and there is no space for new appointments, it is possible that I have to prescribe an extra week of sick leave.”* (Phys6)
- F/B: Occupational physicians have enough time to examine the patient’s situation comprehensively enough / General practitioners do not always have sufficient time: *”In general, I have one hour reserved per appointment in my calendar. It is enough to ask questions and find out about all kinds of things. One has enough time to follow the guidelines recommendations.”* (Phys6) / *“There is enough time for physical examination, but not for going through other things that would inform me about the symptoms and such. Then the patient has to make another appointment. The process prolongs because I did not have time to accomplish it in one go.”* (Phys1)
- B: General practitioners do not always have time to assess patients’ work disability sufficiently: *”One would of course need to understand the nature of each patient’s work, and that takes some time. And then one should have an understanding about the ailment and its link to the work. And this all goes for nothing, if you do not reach a mutual understanding with the patient about the next steps.”* (Phys2)
- F/B: Occupational physicians have enough time to advise patients / General practitioners do not always have time to advise patients sufficiently *”Patients have a lot of questions, and I usually spend some time with these questions at the appointment. There is enough time for that and I find it important.”* (Phys3) / *“It would always be beneficial to discuss these things more with the patient. But if you spend too much time talking with the patient, the schedule is ruined.”* (Phys1)
- F: Physicians have helpful pictures and other material for advising patients: ”*When a patient wants to understand the etiology of the pain, we can look pictures about the anatomy and mechanisms. So he/she can gain an insigt into the possible origins of the pain.”* (Phys8)

Social opportunity
Opportunity afforded by the social and cultural milieu, such as social structures, social support, role models, cultural norms and linguistic/conceptual structures that dictates the way that we think about things (Michie et al. 2011).

Facilitators (F) and barriers (B) described by the target physicians

- F: Physicians can discuss patient-cases with OHS specialists in staff meetings: *"We have a case meeting every week where you can consult colleagues. A physiatrist attends these meetings and we often discuss patient cases concerning pain."* (Phys6)
- F: Physicians can discuss patient-cases with colleagues off the record: *"We often consult colleagues informally. Physicians like discussing the cases with each other and do not always expect any specific answers. It is nice to reflect on these things aloud."* (Phys2)
- F: Senior physicians support readily physicians with less working experience: *"We get a lot of support from seniors. A specialist can be consulted at any time. And there is always enough time to consult colleagues."* (Phys3) *"*
- F: Physicians specializing in occupational health have weekly tutoring sessions with seniors: *"Physicians specializing in occupational health have a tutoring session every week. Problematic patient cases can be consulted there".* (Phys6)
- F: Physicians' work is facilitated by well-functioning multiprofessional co-operation: *"Multiprofessional cooperation is great, and it is functioning here. In a health care center you are quite alone as a physician. Here we have clear assignments, and it supports you."* (Phys7)
- F/B: Physicians share specific professional and cultural norms in the OHS / Commitment to the OHS norms vary among the staff: *"Our mission here is to seek effectiveness. This ideology is guiding us in everything we do. Effectiveness is based on doing things with high quality. Everyone working here, at least in occupational health teams, is committed to this ideology and wants to work here because of it."* (Phys9) / *"I am not sure if common culture is strong, but at least it exists. I would imagine that it affects the majority of people but then for some it does not have any influence at all. They work as they have always done"* (Phys2)
- F: Majority of patients respond positively to physicians' suggestions concerning treatment and/or sick leave: *"There are very few cases where the patient would disagree with me about prescribing antibiotics or sick leave etc."*(Phys3)
- F: Majority of patients respond positively to advice and/or ask questions on own initiative: *"Patients generally accept my advice, and many times they ask ón their own initiative what they are allowed to do and what not. When they are interested they accept the information well."* (Phys4)
- F/B: Many supervisors respond positively to suggested alternatives to full-time sick leave / Supervisors may be reluctant to suggested alternatives to full-time sick leave: *"In my opinion, supervisors are willing to think about solutions. They do not think that it would be better if the worker would stay on sick leave for as long as possible."* (Phys6) / *"There still are supervisors who may have told the patients not to return to work unless fully recovered. This exhausts our options."* (Phys5)

Reflective motivation
Reflective processes involving intentions, plans and evaluations (Michie et al. 2011)

Facilitators (F) and barriers (B) described by the target physicians

- F: Physicians perceive enhancing staying at work/early return to work as important professional goals: *"I feel that my work has an impact and that is why I got interested in it. My work may affect individuals, but the society also. If we manage to keep someone at work for a longer time, it is economically important for the society."* (Phys3)

- F: Physicians perceive pain management as an important part of professional role: *"Pain keeps one from work or is a challenge at work, and of course causes suffering in other ways too. We try to have a holistic approach with patients and not to look only at the eight hours at work. In my opinion, healing pain and suffering fits well with the identity of occupational physicians."* (Phys9)
- F: Physicians perceive advising patients as an important part of professional role: *"As a physician, it is important for me to explain to the patient the mechanisms of the pain and to support his/her own activity."* (Phys7)
- F: Occupational physicians perceive co-operation with supervisors as an important part of professional role*: "Keeping in touch with employers is a part of everyday work. This link between employers and OHS is needed."* (Phys7)
- F: Physicians believe that engaging in recommended behaviors is beneficial for the patients: *"Instead of prescribing sick leaves, supporting return to work is the most important task of a physician. The patient is supported, examined, treated and rehabilitated in order to regain work ability to return to work. If there is some work ability left and the patient is able to carry out a part of his/her work tasks, this can be rehabilitative. We know that prolonging sick leaves have adverse effects.*" (Phys8)
- F: Physicians believe that engaging in recommended behaviors is beneficial for the employer: *"Employers are today under a lot of pressure. I think it is important to support them also by avoiding unnecessary sick leaves. Every pair of hands is needed in working life today. It benefits everbody."* (Phys7)
- B: According to physicians, engaging in recommended behaviors may lead to negative concequences to oneself (e.g. be too burdensome or might reflect badly on the physician): *"A comprehensive doctors certificate is needed for applying partial sickness benefit. It is weird, that applying for full sick leave is simple, but applying for a partial sick leave is very bureaucratic*." (Phys4). *"For me it would be strange to show the guidelines to a patient and say that I use this paper as a ground for my decisions. I base my decisions on my own knowledge and expertise."* (Phys6)
