# Supplementary material for: Evaluation of a guidelines implementation intervention to reduce work disability and sick leaves related to chronic musculoskeletal pain: a theory-informed qualitative study in occupational health care
Source: BMC Musculoskelet Disord. 2022 Mar 22;23:272. doi: 10.1186/s12891-022-05234-8 (PMC8938719; doi:10.1186/s12891-022-05234-8)
Supplement: Supplementary file 4 — Additional file 4. Summary of intervention means applied by the implementers and experienced by the target physicians. The file includes a summary of intervention means applied by the implementers to target the identified barriers to guideline-related behaviours and experienced by the target physicians, with sample quotes. [file 12891_2022_5234_MOESM4_ESM.pdf]

Additional file 4. Summary of intervention means applied by the implementers to target identified barriers to guideline-related behaviours and experienced by the target physicians, with sample quotes

Imp1 = Implementers' interview 1; Imp2 = Implementers' interview 2; Phys1 – Phys9 = Physicians' interviews 1-9

**Applied intervention functions to target identified barriers related to Psychological capability**
**Implementers' aim: Physicians are aware of the OHS guidelines.**<br>Implementers describe applied means to increase target physicians' awareness (Education)<br>• OHS diagnosis-specific guidelines were introduced to physicians and presented later on regularly in staff meetings: *"In January 2016 they (the guidelines) were introduced to all physicians. In every single staff meeting they were brought up."* (Imp1)<br>• Guidelines were (and still are) introduced to new staff as part of their orientation to work in the OHS: *"People who come from somewhere else to work here may have very different practices; they don't know the topic so well. They have to be familiarized (with the guidelines)."* (Imp2)<br>• Physicians with a short-term work contract are not throughly introduced to the guidelines: *"If a physician works here for example for three summer months, we briefly go through the guidelines. But it is not possible to introduce her/him to the whole concept of pain treatment that we apply here."* (Imp2)<br><br>Physicians describe that they have been introduced to the guidelines:<br>• During the launch of the guidelines: *"The staff meetings were mainly used for going through the guidelines. Everybody who worked here at the time surely got to understand the idea behind them."* (Phys2)<br>• When starting working in the OHS afterwords: *"We were several occupational physicians and specialising physicians who started working here at the same time. I think we got an exellent orientation to work. The guidelines were presented to us well."* (Phys7)<br>• No introduction at all (but physicians estimate, however, engaging in recommended behaviors): *"These guidelines have not become familiar to me while working here. But there has been a lot of discussion around musculoskeletal disorders in recent years in general. I see a lot of these patients and apply these recommendations every day."* (Phys6)
**Implementers' aim: Physicians have necessary knowledge and understanding of pain, prevention and treatment of pain, and need for imaging in pain disorders**<br>Implementers describe applied means to increase knowledge and understanding (Education)<br>• Information was delivered in short educational sessions and some coaching sessions led by a pain specialist: "*The education started already before introducing these recommendations. Physicians were mentored by a specialist in pain medicine. The sessions lasted for three hours and there were three of them."* (Imp1) "*Physicians were educated in radiation shielding and the role of x-ray imaging in diagnostics."* (Imp2)<br>• Information was (and still is) delivered in the guidelines: *"The guidelines include information on treatment and what the patient can do him/herself. They guide to paying attention to patient's work, and whether it is possible to modify the work somehow. If pain symptoms seem to persist (two to four weeks) the guidelines check-list is used to detect all factors worsening or prolonging the pain."* (Imp2)<br>• Personal guidance was (and still is) provided to physicians who were noticed by the radiographers to make unnecessary referrals to imaging: *"Our radiological nurses are very active. If they notice that referrals to imaging are made which do not follow the guidelines (e.g. in back pain), they contact us (the management). The referring physician is given feedback for ordering unnecessary x-rays."* (Imp2)

Physicians report that they have received information:<br>• In educational sessions provided by the OHS: *"There were lectures for the personnel given by our own staff and specialists in pain medicine. This topic was discussed continuously."* (Phys2)<br>• From the guidelines: *"It was good to have the check-lists of things you can do, for example, support the patient in continuing at work and modifying work. Or encourage the patient to use his/her hand more actively, for example."* (Phys1)<br>• In OHS staff meetings: *"We have a physiatrist and a psychiatrist working here and we meet them in the case meetings. If they recommend something regarding the care of patients with pain, we follow pretty much their advice."* (Phys6)<br>• From OHS seniors: *"Specialicing physicians meet with tutoring seniors every week for two hours and it is possible to consult them about patient cases and different treatment practices."* (Phys6)<br>• Through learning at work when engaging in new tasks: *"I feel that I have learned many new things through practice. Basic education was so theoretical, and it did not prepare you for practical work. I have been able to utilize that theoretical knowledge now in everyday work"*. (Phys4)<br>• From other sources, such as courses and medias provided for physicians by various Finnish organizations: *"This topic has been discussed a lot in recent years. For example, there have been articles in the Finnish Medical Journal. I have also attended courses on back pain etc."* (Phys5)
**Implementers' aim: Physicians have necessary knowledge of the assessment of work disability and need for sick leave, and the alternatives for full-time sick leave.**<br>Implementers describe applied means to increase knowledge and understanding (Education):<br>• Information was delivered in an e-learning course: *"An e-learning course was prepared on these spesific pain areas. It was available to all physicians in the OHS intranet and they were supposed to study it."* (Imp1)<br>• Information was (and still is) delivered in the guidelines: *"The majority of physicians do not know this subject very well. Now we have a tool for this tricky subject of assessing work disability of an employee with pain symptoms. It is based on scientific evidence, and it is written in plain language."* (Imp1)<br><br>Physicians describe that they have received information:<br>• In an e-learning course provided by the OHS: *"We were supposed to take the e-learning course in prescribing sick leaves." (Phys5)*<br>• From the guidelines: *"I used to check the guidelines to find out about the suitable length of sick leave in musculoskeletal disorders, for example in back or shoulder pain. At least in the beginning, I found the guidelines helpful."* (Phys3)<br>• In staff meetings: *"It came up in the staff meetings, for example, that on an average we described longer sick leaves for shoulder pain than what was recommended. I started to shorten the sick leaves and noticed that it did not cause additional patient visits. Now I prescribe shorter sick leaves."* (Phys4)<br>• Through learning at work from engaging in new tasks: *"I have learned through experience how diseases progress and found out, for example, that a shorter sick leave is often enough."* (Phys4)<br>• From other sources, such as courses and medias provided for physicians by various Finnish organizations: *"Before these guidelines were launched, I used to check the recommendations on the web pages of the Finnish Institute of Occupational Health. And the Finnish Medical Association provided education on prescribing sick leaves."* (Phys8)

Implementers' aim: Physicians remember to consider all relevant factors when making decisions about pain management, work disability, need for sick leave and alternatives for full-time sick leave.

Implementers describe applied means to promote remembering (Enablement)

- Printed guidelines and included check-lists acted (and still act) as prompts for memory: *"The guidelines include the recommendations for sick leaves and the check-list to make sure that everything has been considered at the busy practice. They serve as a reminder for both physician and patient."* (Imp2)
- Physicians were provided with a pain questionnary to be filled with the patients in order to notice patients with a risk of prolonging pain: *"A pain questionnaire was developed to look for factors that predict persistent pain. If one scored above let's say eight points, one was eligible to a pain group."* (Imp2)

Physicians describe helpful reminders:

- Printed guidelines and especially included check-lists: *"In the guidelines there is a check-list to make sure that you have done everything needed. It is good to have a systematic list of possible things to do."* (Phys1)
- Pain questionnary filled with the patients (but not used anymore*): "We used to have pain groups and we used a pain questionnaire to check patients' eligibility to pain groups, but also to chart the "yellow flags". Now that the pain groups do not exist any more, the questionnaire is not used either."* (Phys9)
- Since 2018, an electronic patient record system has guided physicians to take a stand on patient's suitability to alternative work: *"Already last year we wrote in the sickness certificates, if needed, that the patient is eligible for alternative work and also the restrictions. And now it is compulsory to take a stand on alternative work, because you cannot complete the electronic sickness certificate without filling in the related check-box."* (Phys9)

Implementers' aim: Physicians stop to think about their routinized behaviours which may be outdated and in need for change.

Implementers describe applied means to promote reflective practice (Enablement):

- Printed guidelines served (and still serve) as a means for interrupting routinized behaviour: *"Before the new way of thinking is established, it takes quite a lot of effort and one goes easily back to the old ways of doing things. It has to be repeated many times to make one remember to consider all those things. For example, before one prescribes or extends a sick leave, one should go through the check-list and consider different alternatives rather than just doing it automatically or taking the easy way."* (Imp1)

Physicians describe means for assessing the appropriateness of one's practice:

- Guidelines check-lists: *"The guidelines help one to assess if the practices of prescribing sick leave are similar to those of other physicians. The guidelines probably guided me to prescribe shorter sick leaves."* (Phys1)
- Feedback from OHS seniors: *"Specialicing physicians meet regularly with tutoring seniors and have a possibility to get feedback. It would be nice to get more feedback to know where you succeed and what could be done better."*
- Feedback from patients: *"If you first think that a person is able to work, but actually is not, usually nothing terrible happens. He/she reserves another appointment and you get feedback directly from the patient."* (Phys4)
- Information received in educational sessions outside the OHS: *"Continuing education is very important. You have to update your perceptions and practices."* (Phys7)

Applied intervention functions to target identified barriers related to Physical opportunity

Implementers' aim: Physicians have more opportunities to refer patients to non-pharmacological pain treatment

Implementers describe applied means to increase opportunities (Environmental restructuring):

- Pain-groups were set up for patients with chronic pain: *"We also look for alternative treatments. The pain group functioned for quite a while as a tool to support patients' sense of self-efficacy and work ability."* (Imp2)
- Occupational health nurses were educated to discuss and draw up plans for pain management with patients with chronic pain: *"Our nurses were educated to draw up a personal plan for health promotion and pain management for each patient with persistent pain."* (Imp2)

Physicians describe experiences of means for non-pharmacological pain treatment, provided by the OHS:

- According to physicians, pain groups were set up for patients with chronic pain but they are not offered anymore: *"I suppose that there are no pain groups available any more. They were very popular."* (Phys5)
- Physicians refer patients with chronic pain to occupational health nurses: *"I refer many patients to our psychiatric nurse. One often gets an appointment with her sooner than with an occupational psychologist."* (Phys8)

Implementers' aim: Physicians are provided with more time to focus on their special role.

Implementers describe applied means to increase opportunities (Environmental restructuring):

- Occupational health nurses were appointed to take a larger role in pain treatment: "*Nurses were educated in non-pharmacological treatment of pain and physicians got more time to concentrate on matters relevant to their expertise, such as diagnostics, sorting out serious symptoms, medical treatment and, of course, matters that are related to work disability, sick leaves and so on."* (Imp1)

Physicians do not report experiences of increased time to focus on one's special role.

Applied intervention functions to target identified barriers related to Reflective motivation

Implementers' aim: Physicians believe that learning and engaging in recommended behaviors will bring about positive consequences to different stakeholders, including oneself.

Implementers describe means to influence physicians' beliefs (Persuasion):

- Information about the benefits of engaging in recommended behaviours was delivered in educational sessions. *"In order to change your behaviour, you have to understand how it may benefit yourself as a physician, the patients and the employer. We were able to educate the physicians on this topic."* (Imp1)
- Part of the physicians were involved in the formulation of the OHS guidelines, and all physicians were asked to comment the guidelines under way: *"There were four to five of our physicians in the working group making the guidelines. And then there was a psychologist, physiotherapist, occupational nurses and a social worker. All our physicians contributed in the staff meetings. We have discussed the guidelines a lot."* (Imp1)
- Monthly trends of pain-related sick leaves prescribed in OHS were (and still are) presented to physicians in staff meetings: *"The head physician presented new data on sick leaves monthly comparing that month to previous year. We were lucky that the sick leaves in these diagnoses declined rapidly and we were able to present that data."* (Imp1)

- Physicians were acknowledged for adherence to guidelines in staff meetings and by individual e-mails: *”Physicians were also given positive feedback in staff meetings and by personal e-mails that they are doing great job and it shows in the data.”* (Imp1)
- OHS management was committed to the implementation of the guidelines: *”It is very important that the management is committed and takes part in pondering how to implement the guidelines. The physicians need to trust the supervisor to promote and support this.”* (Imp1)

Physicians describe that they have been encouraged to adhere to the guidelines:

- Through seeing the monthly trends of pain-related sick leaves: *”From time to time, led by the head physician, we look at the statistics on sick leaves to see the trends for the whole organisation. It seems that we are heading for the right direction.”* (Phys3)
- Through the enthusiasm shown by the implementers: ”*It is well known that giving information is not enough. The head designer of the guidelines was charismatic enough and showed excitement about this and spread the enthusiasm.”* (Phys2)

Implementers’ aim: Physicians believe that the guidelines allow sufficient professional autonomy

Implementers describe means to influence physicians’ beliefs (Persuasion)

- Information concerning professional autonomy was delivered in educational sessions and staff meetings: *”We informed the physicians that the guidelines address these topics on a quite general level and assist in noticing things that have to be considered. Then it is always between the patient and the physician to ponder what the patient is able to do, and would taking sick leave be helpful or could it even be harmful.”* (Imp1)

According to the physicians, they have much professional freedom:

- Guidelines allow professional autonomy: *”In a way, you can use them (guidelines) to justify the practices to a client. But every time one has to consider the larger picture and the patient’s work. It would be contradictory if we were given exact recommendations, such as to prescribe three days sick leave for back pain*.” (Phys5)
- Lack of surveillance of individual physicians’ practice in the OHS enables professional autonomy: *”I don’t remember being asked about following the guidelines, but we are of course encouraged to do so. I have to admit that surveillance would make no difference.”* (Phys5)
- Physicians do not regard surveillance as necessary: *”I am doing my best to always evaluate the need or suitable length for a sick leave. I don’t think I am too liberal in prescribing sick leaves and then would make an effort to do better knowing that someone is watching me.”* (Phys3)

Implementers’ aim: Physicians have confidence in their capability to have constructive negotiations with patients

Implementers describe means to influence physicians’ beliefs (Education):

- Key messages to be learned were (and still are) provided in the printed guidelines: *”It is easy for the physician to discuss with the patient, for example, the need of a cortisone injection, and to say that based on this up-to-date knowledge of these guidelines it is of no use.”* (Imp2)

According to the physicians, they have gained confidence in negotiations with employees and supervisors:

- Through the printed guidelines: *”Discussing with the patients may be a challenge if they are very worried or expect to get sick leave. When we have these guidelines, it is easier to promote our view of the necessity of a sick leave.”* (Phys9)
- Through accumulating work experience with patients*: ”The job of general practitioners includes a lot of discussing about sick leaves with patients. If they work here for a year, they surely get used to it. But in the beginning, especially if you are a novice, it can be a little annoying.”* (Phys2)
- Through accumulating work experience with supervisors: *”In the beginning, I felt less confident calling the supervisor at the practice, not quite knowing what I could say. But now that I have done it many times and know what to say, it is much easier.”* (Phys6)

e
