# Supplementary material for: Evaluation of a guidelines implementation intervention to reduce work disability and sick leaves related to chronic musculoskeletal pain: a theory-informed qualitative study in occupational health care
Source: BMC Musculoskelet Disord. 2022 Mar 22;23:272. doi: 10.1186/s12891-022-05234-8 (PMC8938719; doi:10.1186/s12891-022-05234-8)
Supplement: Supplementary file 5 — Additional file 5. Summary of factors, beyond physician behaviours, influencing the number of MSD-related sick leave days. The file includes a summary of factors, in addition to physician behaviours, influencing the number of MSD-related sick leave days, as perceived by the OHS professionals, with sample quotes. [file 12891_2022_5234_MOESM5_ESM.pdf]

Additional file 5. Summary of factors beyond physicians' behaviours influencing the number of pain-related sick leave days, as perceived by the OHS professionals, with sample quotes

Imp1 = Implementers' interview 1; Imp2 = Implementers' interview 2; Phys1 = Physicians' interview 1 … Phys9 = Physicians' interview 9; Other prof1 = Other professionals' (occupational physiotherapists, occupational health nurse) interview 1; Other prof2 = Other professionals' interview 2

** Factors are perceived to decrease sick leave days; ** Factors are perecived to increase sick leave days*

Treatment provided by the OHS multi-prosessional personnel (in addition to physicians)

- Patients with minor symptoms are guided to effective self-care by nurses during the assessment of need for treatment: *"If a nurse gives you good advice and you don't need an appointment with a physician or a physiotherapist, that results in fewer sick leaves."* (Phys4) *
- Occupational health nurses provide non-pharmacological care for patients with chronic pain: *"A multiprofessional team solves work disability problems. Nurses see quite a lot of employees with partial work ability. They can coordinate work modifications and organize meetings with supervisors to discuss the modifications."* (Other prof2) *
- Physiotherapists provide effective pain treatment and guide patients in self-care: *"The physiotherapist sees someone who is in pain, examines him/her and gives advice. Most of these people are satisfied and return to work. They don't need new appointments, magnetic resonance imaging or referrals to specialists. They are helped then and there, it is very efficient."* (Imp1) *
- Occupational health psychologists support patients with chronic pain: *"Chronic pain is always somewhat a mental health problem, because pain causes strain and exhaustion, and support of a psychologist is definitely beneficial. Every third Finn with musculoskeletal disorders is depressed as well. Psychologists can use cognitive tools to affect thinking, attitudes, fears and worries."* (Imp1) *
- Full-time physiatrist and psychiatrist serve patients with chronic pain: "*We surely have better resources than many other places. We have access to a psychiatrist and physiatrist."* (Phys5) *
- Shortage of appointment hours for occupational physiotherapists and psychologists: *"There are too few psychologists with respect to the huge workload. Psychologists are a resource that should be utilized more." (Imp1); "Someone with a chronic pain every now and then has to wait for an appointment with a physiotherapist, when there are no free appointments. There are surely too few physiotherapists considering the need. This practice is now well known and used."* (Other prof1) **

Policy, structures and processes in the OHS

- OHS revenue logic allows professionals to focus on the prevention of work disability: *"If occupational health care services focus on preventive work, the need for their services should diminish in the end. However, the revenue logic in the private sector actually goes against focusing on preventive work because they have to produce as high returns to the owners as possible. And this comes from appointments to physicians, diagnostic imaging and operations."* (Imp1) *
- Fairly well-functioning assessment of need for treatment and referral to appropriate professionals: *"Our nurses contact the patients first and assess the need for treatment. They refer patients who seem to have only minor problems to the physiotherapist. Nurses can prescribe sick leave for a few days, and there is no need to visit a doctor for that. I find it a reasonable use of resources. The patient profits as well."* (Phys7) *
- Well-functioning distribution of work among general practitioners and occupational physicians: *"General practitioners see a lot of patients whose problems have nothing to do with work. They can take care of these patients and prescribe sick leave. Without general practitioners there would be masses of patients for occupational physicians."* (Phys4) *

- Self referral to physiotherapists: ”*We also started self referral to physiotherapists, and the physiotherapists were thoroughly briefed for that. They prescribe a lot less sick leaves than physicians. This also resulted in fewer patients with pain problems at the physicians' office.”* (Imp1) *
- Sick leave trends are systematically followed in the OHS and possible setbacks are discussed in staff meetings: ”*Sick leave trends are inspected in staff meetings to see where we are heading. And then we speculate about the findings and is there something we can do?”* (Phys1) *
- OHS actively informs workplaces about new practices concerning e.g., sick leave prescribing: *”We informed the workplaces beforehand through their internal maganize and other medias about our new practices (recommending alternatives to full-time sick leave) and how they benefit the employees. Therefore they were probably prepared for these discussions when they came to see a physician. I suppose that this also affects the sick leave trends.”* (Imp1) *
- OHS personnel actively supports workplaces in using the alternatives to sick leave: *”We know the workplaces very well, so we can give them pretty concrete examples of how they can apply these alternatives. We also have certain topics that we should bring up in meetings with supervisors, and alternative work is on this years' agenda.”* (Other prof2) *

Municipal client organization

- Client organization is committed to enhancing the use of alternatives to full-time sick leaves: *”I think that using alternative work has been one significant factor contributing to the decline of sick leaves. We don't need to prescribe sick leave when people are at work doing alternative tasks.”* (Phys7) *
- Client organization provides employees with services for enhancing health and preventing work disability, e.g., groups supporting healthy behaviours: *”The employer offers masses of different benefits, for example, you can do physical training at an affordable price in places all over the city. And there are groups supporting well-being and work ability.”* (Phys8) *
- Alternatives to full-time sick leave (e.g., work modifications) are difficult to carry out at some duties or workplaces: ”*My patients do mainly physical work. There is no office work available. Sometimes it is possible to clean the storage area or something else. I always ask about the possibilty of alternative work but often it is not feasible.”* (Phys6) **

Employees of the client organization

- Positive cultural change is noticed in most employees' attitudes concerning absence from work: *”It happens that patients themselves suggest alternative work. They have a very positive attitude to this option. They are motivated to work.”* (Phys7) *
- Employees' willingness to avoid absence from work is influenced by current labour market situation and the level of unemployment benefits: *”Patients could indicate that the labour market situation is getting worse and they are afraid to be absent from work. Some patients struggle to stay at work even when they have severe pain if they have already used the maximum days of sick leave with full salary.”* (Phys1); *”The availability of financial benefits is related to this as well. Ample benefits tend to lengthen the sick leaves. In the private sector, the period of full salary is generally shorter.”* (Phys8) */**
- Many employees engage in unhealthy behaviours, which affects work ability in the long run: *”Smoking and using alcohol is common and yet it is known that smoking is a risk factor for MSDs, especially for back pain, as well as sedentary life style and overweight. If people just would take care of their muscle condition and do exercises, it would have a massive impact on sick leaves in physically strenuous work.”* (Phys8) **

Structures and processes in primary and special health care, and social insurance

- Sick leaves are easily prolonging due to insufficient treatment of pain in primary and special health care: ”*Chronic pain gets more expensive to the society than cancer, diabetes and coronary heart disease all together. And most expenses result from work disability. Primary health care does not tackle this and neither does special health care. This would call for entirely different kind of societal debate.”* (Imp1) **
- Long waiting lists to special health care (e.g., in need for operations) extend sick leaves: *”One may need to wait for an operation for a longer time.”* (Phys1) **

- Social insurance regulations hinder the use of part-time sick leave: *”Partial sickness benefit is very handy, but there are inherent barriers to use it. If the physician and the patient at the appointment agree that it would be possible for example to work a half a day and the supervisor agrees as well, the benefit is not available before a full sick leave of two weeks. I call it a compulsory sick leave.”* (Imp1) **
